# Time of *delirium* onset and prognosis amongst Southern Brazilian hospitalized elderly patients

**DOI:** 10.1590/S1980-57642009DN30400007

**Published:** 2009

**Authors:** André Luiz Moschetta, Carine Volkweis Silveira, Roberta Rigo Dalacorte, Rodolfo Herberto Schneider, Irênio Gomes da Silva Filho

**Affiliations:** 1 MD, Institute of Geriatrics and Gerontology, Pontifical Catholic University, Porto Alegre RS, Brazil.; 2 MD, PhD, Institute of Geriatrics and Gerontology, Pontifical Catholic University, Porto Alegre RS, Brazil.

**Keywords:** *delirium*, acute confusional state, elderly, prognostic, hospitalization

## Abstract

The prognostic significance of *delirium* in hospitalized elderly has not yet been fully clarified.

**Objectives:**

The present study was designed to evaluate the relationship between prevalent *delirium* (PrD), incident *delirium* (InD) and final outcome.

**Methods:**

A historical cohort of 261 patients was selected. *delirium* was diagnosed using the Confusion Assessment Method.

**Results:**

The total frequency of delirium detected was 42.5%-31.4% PrD and 16.2% InD. Among patients with InD, the average length of hospital stay was 9.1 days longer than for patients without *delirium* (p=0.002), and the hospital mortality associated with InD was 48% versus 2.7% for those without *delirium* (p< 0.001). However, no difference was observed between patients with PrD and those without delirium.

**Conclusions:**

These results suggest that, when investigating *delirium* and prognosis amongst hospitalized elderly, it is fundamental to differentiate in terms of time of onset. Furthermore, the absence of delirium seems to be an important protective factor.

## Abstract (pt)

O significado do prognóstico de *delirium* em idosos hospitalizados ainda não está completamente elucidado.

**Objetivos:**

O presente estudo foi designado para avaliar a relação entre *delirium* prevalente (DeP), *delirium* incidente (DeI) e o desfecho final.

**Métodos:**

Uma coorte histórica de 261 pacientes foi selecionada. delirium foi diagnosticado pelo Confusion Assessment Method.

**Resultados:**

A freqüência total de delirium foi de 42.5%, DeP 31.4%, e DeI 16.2%. Para pacientes com DeI, a média de duração de hospitalização foi 9.1 dias maior do que aqueles sem *delirium* (p=0.002), e a mortalidade hospitalar associada a DeI foi de 48.3% contra 2.7% dos livres de *delirium* (p< 0.001). Contudo, não houve diferença entre pacientes com DeP e sem *delirium*.

**Conclusões:**

Os resultados sugerem que, ao estudar *delirium* e prognóstico entre idosos hospitalizados, é fundamental diferenciar o problema quanto ao seu momento de início. Além disso, não apresentar *delirium* parece constituir-se num fator protetor importante.

Individuals aged 60 years or older make up approximately 12% of the population in the southern region of Brazil^1^ and represent about 20% of all patients admitted to the regions’ general hospitals.^2-4^ This patient group uses the hospital services more intensively than those of other age groups because their treatment and recuperation takes longer and costs more.^4^ *delirium*, or acute confusional state, is an organic mental syndrome which is acute, transient and fluctuating, that occurs frequently in hospitalized elderly people,^5,6^ and is considered the most common hospital complication in this population.^7-9^

Irrespective of the reason for hospitalization, it is estimated that 15 to 20% of the elderly will be in an acute confusional state on admission, and 25 to 55% will develop the condition during their hospital stay.^7,10^ This frequency depends on the characteristics of the patient and their diseases, on the care dispensed and the sensitivity of the method used for diagnosis.^11^ The link *between delirium* and severity of acute morbid states is well known.^12^ Studies on patients hospitalized in intensive care units, or those suffering from terminal diseases, have demonstrated incidences of the syndrome up to 80%.^10,12,13^

The biological mechanisms involved in the genesis of *delirium* are not yet fully understood. It is known that a complex relationship exists between individual vulnerability and aggressor factors, often multiple, making it more difficult to determine the exact role *delirium* plays in the disease process. Consequently, the data in the literature on the prognostic implications of *delirium* remain controversial. Although various investigative studies, apparently well conducted, have demonstrated an association between this acute confusional state and hospital mortality,^11,14^ prolonged hospital stay,^15,16^ functional decline and greater rates of discharge to places other than the home,^6,7,17,18^ other studies of the same quality have found no such association.^19-23^ It is possible that one of the principal reasons for this difference in results is the failure to distinguish between prevalent delirium (PrD) and incident *delirium* (InD) where this likely leads to the different prognostics.^24,25^ In fact, none of the studies cited above evaluated the differences between the types of *delirium* with respect to the time of onset.

An intensive bibliographic search of the MEDLINE and SCIELO databases, and the respective reference textbooks identified only one paper in which the prognosis of the two presentations of *delirium* were studied separately. In a study of a cohort of 359 patients, McCusker et al. compared the duration of hospital stay amongst elderly patients with PrD versus those with InD, and those without either *delirium*. They found an association of the duration of hospital stay and *delirium* only in the group of patients with InD.^24^ This finding reinforces the hypothesis that PrD and InD contribute differently to prognosis.

The present study was conducted in view of the lack of consensus over the effect on prognosis of *delirium* in elderly patients, the scarcity of data differentiating the time of onset, and the scant information available on its occurrence in our population. The objective was to describe and compare the duration of hospital stay and mortality in elderly patients with PrD versus those with InD, and without either *delirium*, who had been hospitalized in the geriatric ward of a general hospital in the south of Brazil.

## Methods

The study was conducted in a geriatric ward with 16 beds – 8 for use by the Brazilian public health system (*Sistema Único de Saúde - SUS*) and 8 for private patients – in a university general hospital in the south of Brazil. A historical cohort was constructed based on the hospital patient records of all those aged 60 years or older who had been hospitalized consecutively during 2007. We excluded all individuals who had stayed more than 24 hours in another hospital sector (n=102), those that had been discharged for transfer or non-medical reasons (n=3), and those that had been discharged after less that 24 hours of hospitalization (n=2).

These patient files are completed by the medical team of the Geriatric Service, all of whom receive frequent training in how to detect *delirium*. The diagnoses were reached using the Confusion Assessment Method (CAM) which is accurate, precise and valid for this purpose.^26,27^ The criteria used for definition of the cases were: a report of “delirium”, “acute confusional state” or “acute confusional state with organic origin”, or the presence of other information meeting the CAM criteria. Prevalent delirium was defined by a diagnosis of *delirium* in the first 24 hours of admission, and InD when *delirium* was detected after this period. Data was also collected on the demographic details (sex, age and health plan), the cause and characteristics of hospitalization (acute or elective; duration of hospital stay) and on the basic cause of the hospital obituary.

The mean hospital stay and the occurrence of death in the groups with PrD, and without *delirium* on admission, as well as the groups with InD and without *delirium*, were compared. To study the duration of hospital stay, patients who died were excluded from the analysis because we believe the relationship between hospital stay and mortality in these cases may be inverted, if the more seriously ill patients died more rapidly.

The respective research project for this study was approved by the Research Ethics Committee of the São Lucas Hospital at the Pontifical Catholic University of Rio Grande do Sul State. All regulations concerning patient privacy and the handling of the manuscripts were fully respected. In view of the fact that this research was conducted entirely from the patients’ hospital files, informed consent forms were deemed unnecessary.

To compare the frequencies of PrD and InD according to demographic and clinical variations, and mortality, the basic chi-squared test was used, while for the age groups the chi-squared trend test was applied. In the analysis of mortality, Fisher’s exact test was used whenever an expected value of less than five was found. The comparison of mean hospitalization times of the groups with and without *delirium*, either prevalent or incident, was performed using Student’s t test. The significance level was assumed as 5%. The data was analyzed with the SPSS software package version 11.5.

## Results

Table 1 lists the demographic and clinical data of the 261 patients analyzed in this study. Patient ages ranged from 60 to 104 years, having a mean of 79.9 years (SD=8.4). The predominant characteristics were female gender (58.4%), more than 80 years old (54.8%), public health system patients (53.3%), and suffering from acute disease (72.5%). The most frequent reasons for hospitalization were elective clinical indication (22.2%) followed by respiratory infections (12.3%). The mean length of hospital stay was 13.2 days (SD=11.2), and 32 patients (12.3%) evolved to hospital death.

**Table 1:** Time of *delirium* onset, demographic and clinical characteristics.

	N (%)	Prevalent delirium*				Incident *delirium*		
		N	%	p^†^		N	%	p
Sex				0.202				0.681
Male	106 (40.6)	38	35.8			12	17.6	
Female	155 (59.4)	44	28.4			17	15.3	
Age group				0.020				0.369
60-69	28 (10.7)	4	14.3			4	16.7	
70-79	90 (34.5)	26	28.9			7	10.9	
80 or older	143 (54.8)	52	36.4			18	19.8	
Health Plan				0.075				0.532
Public	139 (53.3)	37	26.6			15	14.7	
Private	122 (46.7)	45	36.9			14	18.2	
Type of hospitalization				<0.001				0.577
Acute	190 (72.8)	81	42.6			19	17.4	
Elective	71 (27.2)	1	1.4			10	14.3	
Reason for hospitalization				<0.001				0.318
Elective clinical^‡^	58 (22.2)	1	1.7			7	12.3	
Respiratory infection	56 (21.5)	30	53.6			5	19.2	
Urinary infection	23 (8.8)	17	73.9			3	50.0	
Other infection	19 (7.3)	3	15.8			4	25.0	
Acute renal insufficiency	15 (5.7)	7	46.7			1	12.5	
Heart disease^§^	13 (5.0)	2	15.4			0	0.0	
Elective surgery	13 (5.0)	0	0.0			3	23.1	
Collateral medication effect	7 (2.7)	5	71.4			0	0.0	
Acute abdominal	7 (2.7)	4	57.1			0	0.0	
Other	50 (19.2)	13	26.0			6	16.2	
Total	261 (100)	82	31.4	--		29	16.2	--

* Prevalent *delirium*: proportion of *delirium* diagnosed within the first 24 hours of Hospital admission. Incident *delirium*: proportion of *delirium* diagnosed during hospital stay in patients who were *delirium*-free on arrival;

† Values obtained from the χ^2^ test. For the variable age, the χ^2^ trend test was used;

‡ Includes: non-surgical diagnostic investigation, therapeutic organization, or family exhaustion;

§ Includes: acute decompensated heart failure, cardiac arrhythmia, acute coronary syndrome, or stroke.

The total incidence of *delirium* was 42.5%, with PrD occurring in 31.4%, and InD in 16.2%. The incidence of PrD differed according to age with a tendency to rise with increasing age and to be higher in the group hospitalized in the private patients’ beds. (36.9% v. 26.6%, p=0.075). The proportion of PrD in patients hospitalized for acute conditions was higher (42.6% v. 1.4%, p< 0.001). With regard to the reason for hospitalization, PrD was more frequent in cases of urinary infection (73.9%), collateral medication effect (71.4%), acute abdominal surgery (57.1%) and respiratory infection (53.6%). No statistically significant difference in InD frequency was found that could be traced to the demographic and/or clinical characteristics.

The duration of hospital stay and mortality rate was the same in patients with and without PrD (Table 2). In patients with InD, however, the mean length of hospital stay was 9.1 days, and mortality was 48.3%, significantly greater than in the individuals without *delirium* who presented a mean hospital stay of 1.2 days (p=0.002) and a mortality rate of 2.7% (p< 0.001).

**Table 2:** Time of *delirium* onset, duration of hospital stay and mortality.

	Prevalent *delirium*		p
	**Yes**	**No**	
N	82	179	0.602*
Duration of hospital stay in days, of elderly patients who were discharged (mean±SD)	12.3±9.9	13.1±11.0	
Mortality N (%)	14 (17.1)	18 (10.1)	0.109^†^
	**Incident *delirium***		**p**
	**Yes**	**No**	
N	29	150	0.002*
Duration of hospital stay in days, of elderly patients who were discharged (mean±SD)	21.3±11.0	12.2±10.7	
Mortality N (%)	14 (48.3)	4 (2.7)	<0.001^‡^

* t test;

† Chi-squared test;

‡ Fisher's exact test.

## Discussion

Research on *delirium* is subject to innumerable methodological problems due to the lack of clarity surrounding its physiopathology and the lack of consensus with respect to the criteria for diagnosis. Notwithstanding these issues, the results of the present study as regards the frequency of the incidence of *delirium*, and the association of PrD with older age, acute care hospitalization, and the reasons for hospitalization all agree with the data in the literature.^7,15,17,22,28^ The greater prevalence of *delirium* found in patients with urinary infections and collateral medication effects can be explained by the fact that the signs and symptoms involved with these conditions are less apparent amongst the elderly, delaying diagnosis and increasing the chances of developing acute confusion states before treatment is initiated.

We found no statistically significant association between PrD and prognosis in spite of the greater mortality in the group that presented *delirium* on admission. It is possible that our sample was too small to demonstrate any difference. Also, we excluded those patients who had been spent more than 24 hours outside the geriatric ward where these patients would generally be in a more serious condition, and would probably have presented with *delirium* on admission and have a poorer diagnosis. *delirium* that initiated during hospitalization, however, was strongly associated with both the duration of hospital stay and mortality. This difference between PrD and InD was first described by Mc Cusker et al., who found longer periods of hospitalization in patients with InD and no association of PrD with prognosis.^24^

The difference found in the prognostic values of PrD and InD suggests that PrD is not necessarily an indicator of serious illness, and may be more closely related to previous vulnerable states (for instance: advanced age, cognitive impairment, institutionalization, fragility) which facilitate the onset of a confusional state. This neurological fragility likely influences the outcome but to a lesser extent than the degree of severity of acute disease. On the other hand, patients that developed InD, but were admitted to the hospital problem-free generally demonstrated less vulnerability. The occurrence of InD in these patients would indicate, therefore, negative evolution of the conditions that led to hospitalization in the first place, or occurrence of a hospital-acquired complication.

Clinical, epidemiological and basic scientific evidence supports the hypothesis that *delirium* is merely an indicator of prognosis and is not itself a cause of a poor prognosis.^11,18,29^ The significance of *delirium* may lie in its ability to reveal a deleterious interaction between the individual and the aggressor factor. In some cases, increased individual vulnerability could lead to a greater occurrence of PrD, less relevant for the prognosis. In other cases, because of more aggressive disease, which is generally the cause of the appearance of InD, the prognosis is worse. Considering the greater prevalence of PrD (31.4% v. 16.2%), it seems that increased individual vulnerability is more frequent in elderly patients with acute disease. This is clearly evident in cases of *delirium* due to benign problems, such as fecal impaction or mild dehydration which, in the majority of cases, would not justify hospitalization, much less lead to a prolonged hospital stay or a greater rate of mortality.

The mortality among the *delirium*-free group was much lower in comparison to patients who presented *delirium* (2.7% v. 25%) at some point, and this result is in line with findings of other studies.^11,17^ Therefore, the absence of *delirium* appears to indicate some degree of prognostic protection rather than the opposite. This finding may help to reach decisions in complicated situations where there is doubt over indicating invasive interventions in hospitalized elderly patients. The absence of acute confusional state in the patient’s file would thus indicate a good prognosis, or less individual vulnerability, and consequently, lower risk of unfavorable evolution.

First recognizing that amongst elderly hospitalized in geriatric wards, PrD is more related to an increased state of individual vulnerability (which has little effect on the prognosis), and that secondly, the occurrence of InD is most likely due to a serious situation (indicating evolution to worsened state), can help to clarify the role played by *delirium* as an indicator in prognosis – although this matter remains controversial. We believe, however, that it is fundamental to differentiate the time of *delirium* onset. In addition, the absence of *delirium* seems to be an important protective factor that could assist decision making regarding diagnostic and therapeutic procedures in hospitalized elderly patients. However, this hypothesis requires further elucidation.
